# P20 Rapid 15 min test to identify the three most common Gram-negative uropathogens in urinary tract infections

**DOI:** 10.1093/jacamr/dlad077.024

**Published:** 2023-08-02

**Authors:** Sylvain Rama, Mathew Kyriakides, Henry Newman, Kabir Bonney-Bhandal, James Preece, Jean-Baptiste Vendeville, Neciah Dorh, Josephine Dorh

**Affiliations:** FluoretiQ Limited, Unit DX, St Philips Central, Albert Road, Bristol BS2 0XJ, UK; FluoretiQ Limited, Unit DX, St Philips Central, Albert Road, Bristol BS2 0XJ, UK; FluoretiQ Limited, Unit DX, St Philips Central, Albert Road, Bristol BS2 0XJ, UK; FluoretiQ Limited, Unit DX, St Philips Central, Albert Road, Bristol BS2 0XJ, UK; FluoretiQ Limited, Unit DX, St Philips Central, Albert Road, Bristol BS2 0XJ, UK; FluoretiQ Limited, Unit DX, St Philips Central, Albert Road, Bristol BS2 0XJ, UK; FluoretiQ Limited, Unit DX, St Philips Central, Albert Road, Bristol BS2 0XJ, UK; FluoretiQ Limited, Unit DX, St Philips Central, Albert Road, Bristol BS2 0XJ, UK

## Abstract

**Background:**

Every year, 6.2 million cases of urinary tract infection (UTI) are reported in England and Wales and have the second highest antibiotic prescription rate. The absence of fast and sensitive diagnostic tests has increased empirical antibiotic use and has contributed to the rise of antibiotic-resistant infections. To address this challenge, we have developed NANOPLEX^™^: a rapid 15 min test that uses glycan-functionalized-latex nanoparticles, microscopy and image-analysis software to identify and enumerate samples with bacteria. In this work, we show that NANOPLEX^™^ can correctly identify the three most common uropathogens using lab reference strains, clinical isolates and clinical urine samples.

**Materials and Methods:**

The NANOPLEX^™^ assay was performed as per Vendeville *et al.* 2021.^1^ ACS Biomater 2022; 8, 1:242-252. Clinical isolates of *Escherichia coli*, *Klebsiella pneumoniae* and *Proteus mirabilis* were obtained from an NHS clinical microbiology lab waste stream. *N*=30 isolates (per species) were tested via single-blind study protocols at concentrations of 10³–10^8^ cfu/mL and compared with microbial culture to define performance metrics (sensitivity, specificity, PPV, NPV and accuracy) of each probe.

**Results:**

A total of 90 isolates were tested against their target probes and demonstrated >90% accuracy (sensitivity 88%–90%, specificity 94%–98%, PPV 93%–98%, NPV 88%–90%, accuracy 91%–94%) for all probe-bacteria combinations at clinically significant concentrations (10^5^ cfu/mL).

**Conclusions:**

NANOPLEX^™^ was able to successfully detect the top three common Gram-negative pathogens in UTI at clinically relevant concentrations. A compact hardware and single-use cartridge system is under development to enable urologists to conduct rapid and accurate UTI testing in point-of-care environments. Larger clinical studies are planned to clinically validate these results.
